# Neonatal ischemic limb lesions: From etiology to topical nitroglycerine. A case series analysis

**DOI:** 10.1111/dth.15426

**Published:** 2022-03-12

**Authors:** Raffaele Falsaperla, Manuela Lo Bianco, Andrea Giugno, Germana Lena, Laura Sciuto, Francesco Spata, Claudio Guarneri, Piero Pavone, Martino Ruggieri

**Affiliations:** ^1^ General Pediatrics and Pediatric Acute and Emergency Unit, Policlinico “G. Rodolico‐San Marco” University Hospital University of Catania Catania Italy; ^2^ Neonatal Intensive Care Unit, Policlinico “G. Rodolico‐San Marco” University Hospital University of Catania Catania Italy; ^3^ Post Graduate Programme in Paediatrics, Department of Clinical and Experimental Medicine University of Catania Catania Italy; ^4^ Neonatal Intensive Care Unit “Giovanni Paolo II” Hospital Ragusa Italy; ^5^ Department of Biomedical and Dental Sciences and Morphofunctional Imaging, Section of Dermatology, A.O.U.P. “Gaetano Martino” University of Messina Messina Italy; ^6^ Department of Clinical and Experimental Medicine, Section of Pediatrics and Child Neuropsychiatry University of Catania Catania Italy; ^7^ Unit of Rare Diseases of the Nervous System in Childhood, Department of Clinical and Experimental Medicine, Section of Pediatrics and Child Neuropsychiatry University of Catania Catania Italy

**Keywords:** ischemic lesions, newborn, NICUs, nitroglycerine, side effects

## Abstract

Although rare, ischemic lesions in neonates may occur in Neonatal Intensive Care Units (NICUs) secondary to routine procedures and/or medicaments. We present double‐center case series, reporting three preterm neonates with ischemic lesions following cardiac arrest and radial blood sampling. The overall outcome after treatment with 2% nitroglycerine (NTG) ointment showed optimal results with no adverse events. The most frequent causes responsible for the onset of such lesions are peripheral arterial catheterization procedures and dopamine extravasation. Our series describe the cardiac arrest as an underestimated cause of onset. Despite the optimal results emerging from the treatment of such lesions with NTG ointment, both in our experience and in the scientific literature, a defined protocol for its use in NICUs is not currently available, hence the need for further studies.

## 1 INTRODUCTION

Ischemic skin lesions of the extremities in neonates are exceptionally rare.^1^ They have been frequently associated with catheterization maneuvers or dopamine administration,^2^ and several risk factors can ease their occurrence, (e.g., hypercoagulable state and sepsis).^3^, ^4^ Nevertheless, in many cases a specific cause remains unidentified.

We present three cases of neonatal ischemic lesions, respectively occurred following cardiac arrest and arterial blood sampling, successfully treated with 2% nitroglycerine (NTG) ointment.

Considering the optimal results reported in the literature and according to our experience, the aim of this report is to highlight the need for a defined protocol in the use of NTG, which could better describe doses, modality and timing for application, to avoid any potential adverse effects.

## 2 CASE SERIES

This is a double‐center case study, including the Neonatal Intensive Care Units (NICUs) of “San Marco” (Catania) and “Giovanni Paolo II,” (Ragusa) hospitals, Sicily (Italy). Informed consent was obtained for each case.

The first two cases are twins (Case 1 and 2) delivered by c‐section at 27 GA because of fetal heart rate decelerations. The mother was 34 year‐old, gravida 1, para 0, abortion 0. She received two doses of betamethasone for preterm labor (Tables 1 and 2).

**TABLE 1:** Cases reported in our series listed by gender, weight at birth, gestational age, delivery mode, and APGAR

	Gender	Weight at birth (g)	GA (days)	Delivery	APGAR (1′–5′)
Case 1	M	760	189	C‐section	4–7
Case 2	F	650	189	C‐section	4–4
Case 3	F	620	171	C‐section	6–8

**TABLE 2:** Cases reported in our series of preterm newborn treated with 2% NTG ointment, listed by post‐natal age (PNA), causes of ischemia, mother's risk factors, localization of lesions, treatment of lesions, outcome and adverse effects

	PNA, (days)	Cause of ischemia	Mother's risk factors	Ischemic site	Treatment	Outcome	Adverse events
Case 1	1	Cardiac arrest	HBsAg +	Feet and the right hand	HBIG and hepatitis B vaccine; antibiotics; 2% NTG ointment	Complete recovery after 96 h; died for cardiac arrest	No
Case 2	1	Cardiac arrest	HBsAg +	Four limbs extremities	HBIG and hepatitis B vaccine; antibiotics; RBC and PLT transfusion; 2% NTG ointment	Complete recovery after 24 h; died for cardiac arrest	No
Case 3	7	Post‐arterial blood sampling vasospasm	Placental abruption, gestational hypertension	Right forefinger	Galenic 2% NTG ointment	Complete recovery after 24 h	No

Abbreviations: HBIG, human hepatitis B immunoglobulin; NTG, nitroglycerine; RBC, red blood cells; PLT, platelets.

### 2.1 Case 1

**FIGURE 1:**
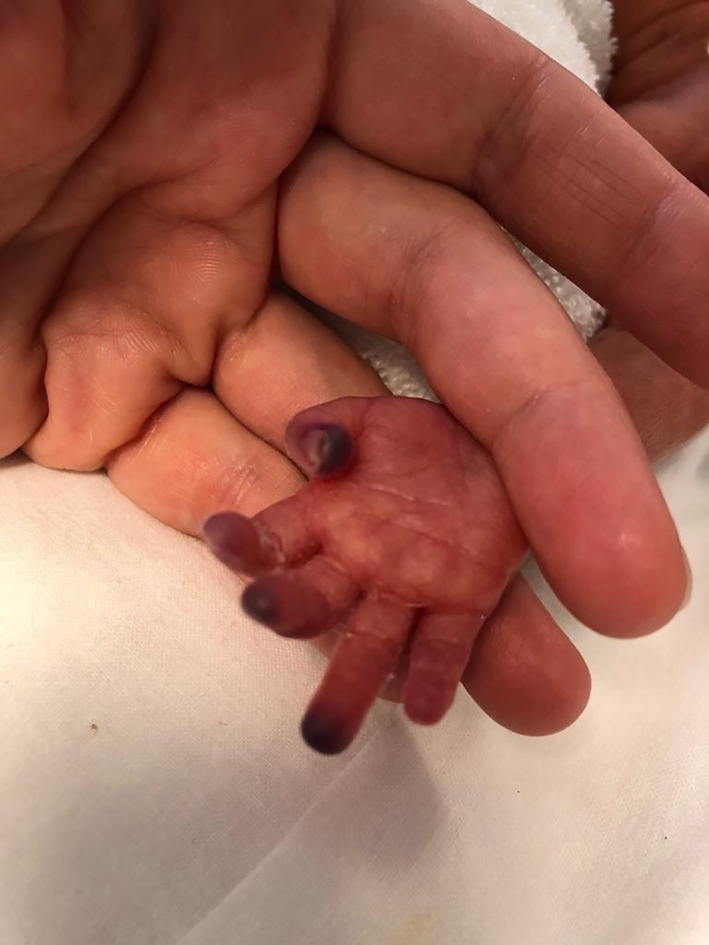
Ischemic lesions of the right hand fingertips following cardiac arrest in a 27 weeks preterm twin

**FIGURE 2:**
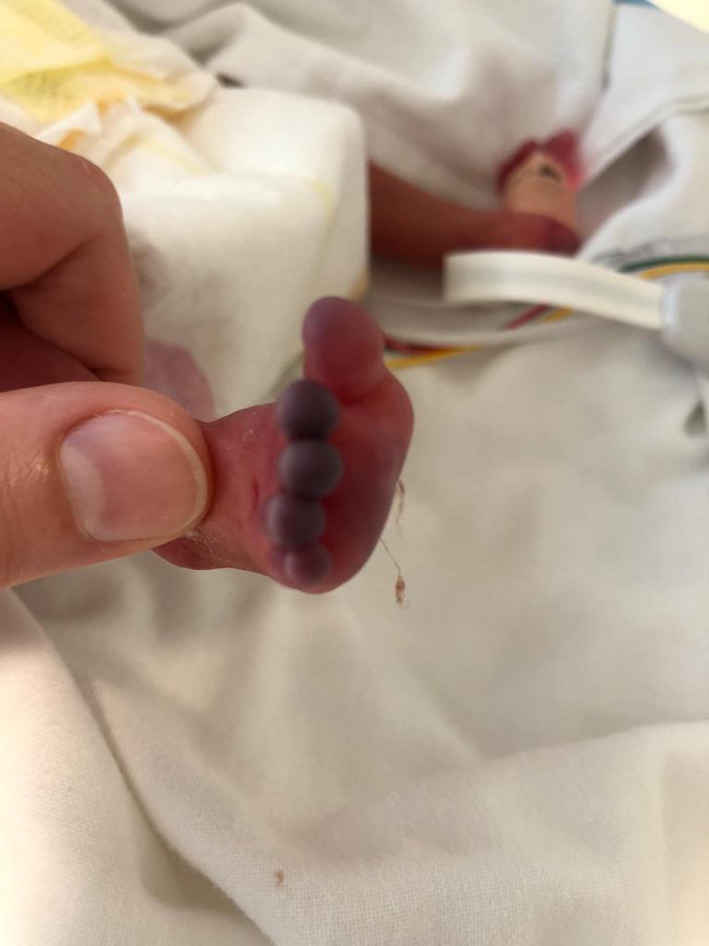
Ischemic lesions of the foot following cardiac arrest in a 27 weeks preterm twin

The first‐born was a 760 g‐infant. His APGAR was 4 and 7 at 1′ and 5′ min, respectively. The patient showed a respiratory distress syndrome (Silverman score: 8). He was intubated and treated with INSURE. A Non‐Invasive Ventilation was adopted and umbilical arterial line (UAL) was inserted. At 12 h of life, due to desaturation and bradycardia crisis, cardiopulmonary resuscitation (CPR) was performed. Three hours later, ischemic lesions involving the extremities of feet and the right hand were gradually evolving (Figures 1 and 2). Dorsalis pedis pulses were no more palpable; the radial pulses were weak. Doppler‐ultrasound showed hypoperfusion, without thrombi. 2% NTG ointment was applied every 6 h.

After 72 h, the improvement of lesions was evident and the resumption of pulses was confirmed at the color‐doppler. At 96 h the affected limbs showed normal color, with total regression of the hypoperfusion.

At day 10 of life, due to the severity of clinical conditions the baby went to cardio‐circulatory arrest and death.

### 2.2 Case 2

The second twin was a female 650 g‐infant. APGAR score was 4 and 6 at 1′ and 5′. She was intubated and endotracheal surfactant was administered. PC‐CMV ventilation was initiated, following the insertion of an UAL. At 15 h of life, she had a cardiovascular arrest; CPR was performed for 6 min with resumption of vital parameters. Few hours later, ischemic skin lesions appeared to the extremities of all limbs. Her radial and femoral pulses were no more appreciable and a color‐doppler examination showed hypoperfusion without thrombi. 2% NTG ointment was applied every 6 h until resolution of lesions.

Compared to her twin, she showed an earlier response to the treatment in about 24 h, however cardiac arrest occurred once more, this time with no resumption from CRP.

### 2.3 Case 3

**FIGURE 3:**
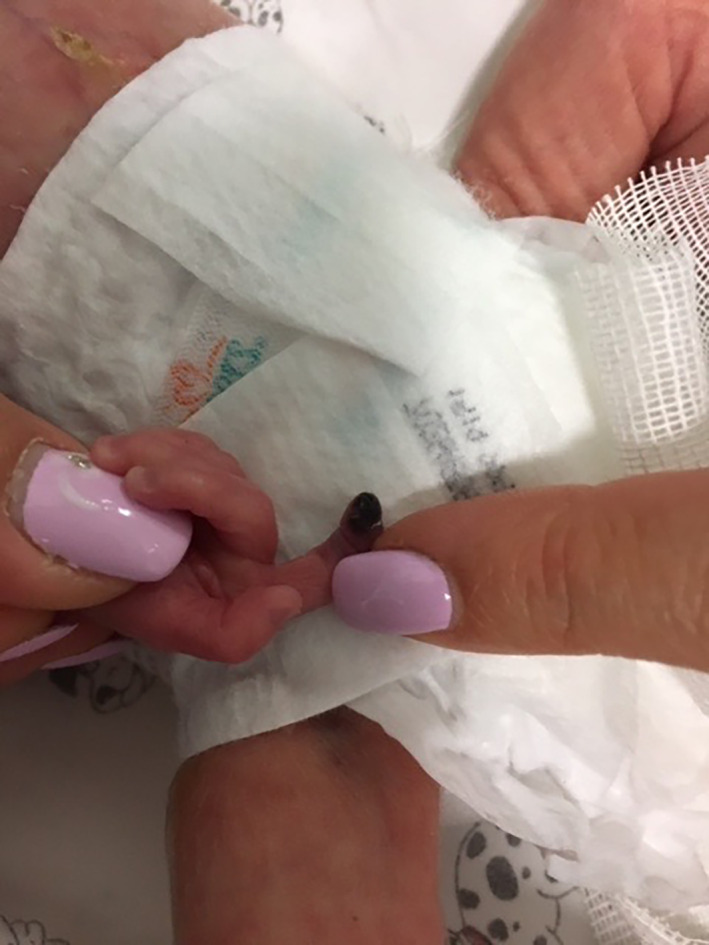
Ischemic lesion appearing in the index finger of the right hand of a 23 + 3 weeks newborn after arterial blood sampling

The third case was a 620 g‐female infant. At 23 + 3 weeks of gestation, a placental abruption led to an emergency c‐section. APGAR score was 6 at 1′ and 8 at 5′. She presented a rosy skin color, with widespread ecchymosis on her limbs. Due to a cardiac arrest, CRP was made and then n‐CPAP ventilation (FiO2 30%) was administered. She presented intercostal retractions, diaphragmatic insertion, and tachypnoea (Silverman score 4); therefore, she was intubated and treated with mechanical ventilation (SIMV‐ET). Her general conditions progressively improved, but at the 7th day of life, after a blood sampling performed at the radial artery of her right arm, an ischemic lesion occurred in the index finger (Figure 3), probably due to a vasospasm. A galenic 2% NTG ointment, (composed by 50 mg of NTG, 25 g of white vaselin and lactose q.s.), was applied every 8 h. No adverse events were noticed and after 7 h the involved area showed total regression of the lesion.

## 3 DISCUSSION

The onset of localized ischemic lesions is a rare condition in neonates, occurring when a causal factor leads to a reduced/absent perfusion of a skin area. They appear as a skin discoloration, sometimes combined with atrophy, ulceration, and finally necrosis. Several predisposing factors can ease their onset: maternal/neonatal hyper‐coagulable state, amniotic bands, abruptio placentae, sepsis, congenital heart disease, gestational diabetes, and hypertension.^5^, ^6^, ^7^

The most frequent conditions associated with the onset of ischemic lesions are catheterization procedures and dopamine administration.^4^ Dopamine (3,4‐dihydroxyphenylethylamine) is a vasoactive drug, whose increasing doses progressively stimulate dopaminergic, alpha‐ and beta‐adrenergic receptors. It is commonly used in NICUs for severe hypotension and shock. Nevertheless, its extravasation can induce vasoconstriction due to its alpha‐adrenergic effect, thus ischemic lesions may appear.^8^

The onset of ischemic lesions following cardiac arrest, has never been described before.

In our cases the cardiac arrest represents the most probable cause of ischemic lesions since catheter placement procedures were successful, no other causative treatment (e.g., dopamine) were administered at that time, and the risk factors to consider (e.g., prematurity) were present from birth. Therefore, the well‐known restriction of peripheral circulation, its deviation to noble organs, and the following heart failure may clarify the onset of ischemic lesions involving all the extremities. Noteworthy that in cases of ischemic lesions due to catheter placement, they are relative to the anatomical part supplied by the artery involved.

On the contrary, arterial blood sampling and vascular catheterization are common procedures in NICUs, and obstructions of blood flow due to the onset of thrombi, emboli or vasospasm are frequent complications.^9^, ^10^, ^11^ They may result from endothelial damage caused by the introduction of the catheter, exposing collagen and other subendothelial tissues to the action of coagulation factors. Vasoconstrictor molecules determine vasospasm and ischemia, leading to complete obstruction and necrosis.^12^ Generally, removal of the vascular catheter, elevating the affected limb and warming the contralateral one resolves ischemia. In about 0.5% of cases, ischemia persists, and drug treatments are required.^13^

When clot formation arises from iatrogenic vascular traumas, the use of thrombolytic agents or heparin has shown low efficacy. Moreover, these treatments are not indicated in the neonatal population due to the potential risk of hemorrhage.^9^

The administration of NTG (1,3‐dinitrooxy‐ propane‐2‐ylnitrate) was first described in 1847 as treatment of angina pectoris, which still remains its most notable clinical use.^14^ NTG is known to be the most powerful vasodilator, thus its pharmacological properties have found application in several clinical contests: Raynaud disease, venepuncture, aid to catheter insertion, survival of skin flaps following plastic surgery, severe cutaneous purpura.^9^, ^15^, ^16^, ^17^, ^18^, ^19^, ^20^ After more than a century, the specific NTG mechanism of action still remains a dark spot in the pharmacology knowledge. Apparently, the mitochondrial enzyme “aldehyde dehydrogenase” is responsible for the NTG conversion to nitrous oxide (NO), which activates the guanylate cyclase, finally leading to the relaxing of the smooth vascular muscles, thus inducing venous and arterial dilatation in 30–60 min.^21^ Therefore, NTG definitely increases the collateral circulation of localized areas of peripheral ischemia. The effects usually last 6–8 h and adverse events include local erythema, methemoglobinemia, headache, hypotension, and dizziness.^22^ Apart from the sublingual administration, it is even absorbed through the skin, releasing the highest concentration below the point of application, hence the ratio of its topical use in ischemic lesions.^5^, ^23^, ^24^

Concerning neonatal age, in 1988 O'Reilly et al. first described the application of a patch containing glyceryl trinitrate 25 mg in a baby who developed ischemic lesion of the foot following the extravasation of intravenous parenteral nutrition. At the time of patch removal, the beneath skin appeared well perfused and healed without scarring, while the not covered surrounding area became necrotic.^25^ One year later, Denkler et al.^8^ applied 2% NTG ointment in a 34‐week‐old neonate, following dopamine extravasation injury of the hand and the foot, reporting optimal outcome and definitely recommending its use. Wong et al.^9^ were the first in 1992 to report the use of NTG in neonatal tissue ischemia due to radial artery catheterization, which became through the years its most representative field of application in newborns.

In our series, the treatment with 2% NTG gave full recovery in all cases. No side events were recorded.

A recent systematic review by Sushko et al.,^26^ including 23 articles (case reports, case series, and retrospective audit) about newborns treated with NTG ointment, patch or spray concluded that NTG ointment represents a safe therapeutic modality for neonatal tissue ischemia, although its production in Canada was discontinued in July 2018. Luckily, to date, in Italy NTG ointment, or at least its galenic preparation, is easily disposable. However, to prevent the same discontinuation from happening, it would be worthwhile to realize a protocol of NTG ointment practical use in neonatal care, which is not currently available. The effective dosage and duration of treatment, indeed, still remain not established. Nevertheless, in order to avoid systemic vasodilation in infants, the most widespread dosage reported in literature is of 4 mm/kg, equivalent to 0.2–0.5 μg/kg per minute when given intravenously.^9^ Concerning the duration of treatment, it varies from 3 h to 27 days, depending on the clinical response: usually the discontinuation is considered when signs of perfusions, (e.g., skin discoloration resolution) clearly appear.^3^

Based on our experience and on the scientific literature so far reported, we strongly recommend the application of 2% NTG ointment as soon as possible when the ischemic lesions appear, referring to the upon reported dosage of 4 mm/kg. The application of ointment can be repeated after 6/8 h if lesions reappear or fail to resolve. We do not advise on NTG patch or spray since the scientific evidence at this point is extremely limited^26^ and we have no direct experience of using this alternative options. However, we believe that their use does not allow adequate adaptability of doses and respect of boundaries of the lesions. Finally, we suggest the discontinuation of treatment exclusively for critical adverse events, (e.g., methemoglobinemia and severe hypotension) but not for transient episodes of mild low blood pressure since they tend to resolve spontaneously.^9^

## 4 CONCLUSIONS

NTG has shown optimal results in the treatment of neonatal ischemic lesions. Its pharmacologic properties have proven to be effective for lesions with different causes with minimal or absent side effects.

In spite of the demonstrated success, to date only few cases are reported. Moreover, the lack of RCTs does not allow neonatologists to routinely consider NTG in case of ischemic lesions, preferring instead other therapies (e.g., heparin), which have not proven to be as effective.

The cardiac arrest is another possible cause of ischemic lesions, mainly if all extremities are interested, not to be neglected and never reported before, for which NTG may be as effective as reported for other conditions, paving the way to a wider field of application in neonatal care.

## CONFLICT OF INTEREST

The authors declare that there is no conflict of interest.

## AUTHOR CONTRIBUTIONS

Raffaele Falsaperla: conceptualization, methodology, project administration; Manuela Lo Bianco: writing, reviewing and editing; Andrea Giugno: data curation, writing, original draft preparation. Germana Lena: visualization, investigation. Laura Sciuto: literature searching, designing; Francesco Spata: conception, resources; Claudio Guarneri: reviewing and editing; Piero Pavone: resources, revising; Martino Ruggieri: supervision, project administration.

## Data Availability

Data sharing not applicable to this article as no datasets were generated or analysed during the current study.
